# Efficacy of Overtube in Preventing Aspiration Pneumonia During Esophageal Endoscopic Submucosal Dissection

**DOI:** 10.1002/deo2.70400

**Published:** 2026-08-11

**Authors:** Masaki Ominami, Shusei Fukunaga, Daiki Kitagawa, Mitsuhiro Kono, Shunsuke Takahashi, Tatsuya Kurokawa, Yoshinori Shimamoto, Yuki Hisaki, Natsumi Maeda, Yuki Ishikawa‐Kakiya, Kojiro Tanoue, Akinari Sawada, Yu Nishida, Hirotsugu Maruyama, Yuji Nadatani, Koji Otani, Shuhei Hosomi, Yasuhiro Fujiwara

**Affiliations:** ^1^ Department of Gastroenterology Osaka Metropolitan University Graduate School of Medicine Osaka Japan

**Keywords:** adverse events, aspiration pneumonia, early esophageal cancer, endoscopic submucosal dissection, overtube

## Abstract

**Objectives:**

Aspiration pneumonia is a potential adverse event of esophageal endoscopic submucosal dissection (ESD). Using an overtube during esophageal ESD may reduce the amount of fluid refluxing into the oral cavity and prevent aspiration pneumonia. This study aimed to clarify the preventive effects of overtubes on aspiration pneumonia during esophageal ESD.

**Methods:**

Between January 2015 and May 2020, 534 consecutive patients underwent esophageal ESD under intravenous anesthesia without endotracheal intubation at our hospital. Using propensity score matching and inverse probability of treatment weighting (IPTW), we retrospectively evaluated the risk factors and incidence of aspiration pneumonia during esophageal ESD.

**Results:**

A total of 393 patients who underwent esophageal ESD were enrolled. After matching, 99 matched pairs of patients either had an overtube used during esophageal ESD or did not. The overall incidence of aspiration pneumonia was 15.2%. The incidence of aspiration pneumonia was significantly higher in the non‐overtube group than in the overtube group (22.2% vs. 8.1%; *p* = 0.009). Multivariate logistic regression analysis showed that non‐use of an overtube was an independent risk factor for aspiration pneumonia (odds ratio [OR], 4.21; 95% confidence interval [CI], 1.66–10.65; *p* = 0.002). After adjustment with IPTW, non‐use of an overtube also increased the risk of aspiration pneumonia (OR, 3.76; 95% CI, 1.54–9.21; *p* = 0.004).

**Conclusions:**

Not using an overtube was a risk factor for aspiration pneumonia during esophageal ESD. Therefore, the use of an overtube is recommended to prevent aspiration pneumonia.

**Trial Registration:**

N/A.

## 1 Introduction

In recent years, advances in diagnostic imaging have made it possible to detect esophageal cancer at an early stage [1]. Endoscopic submucosal dissection (ESD) for early esophageal cancer is widely used as a minimally invasive treatment with a high degree of curative potential [2, 3, 4, 5, 6]. While the main adverse events of esophageal ESD include bleeding, perforation, and strictures, aspiration pneumonia can also occasionally occur [7, 8].

Compared to the major adverse events of esophageal ESD mentioned above, aspiration pneumonia has received less attention and has been reported less frequently, with its incidence reported to be 1.6%–32.0% [8, 9, 10]. Furthermore, there are few reports on methods to prevent aspiration pneumonia following esophageal ESD [9]. During esophageal ESD, fluid tends to reflux into the oral cavity, which increases the likelihood of aspiration pneumonia. An overtube, which is inserted through the mouth into the cervical esophagus, facilitates smooth insertion and removal of the endoscope during upper gastrointestinal ESD. The use of an overtube during esophageal ESD may reduce the amount of fluid refluxed into the oral cavity and prevent aspiration pneumonia.

Therefore, this study aimed to clarify the preventive effects of overtubes on aspiration pneumonia during esophageal ESD.

## 2 Methods

### 2.1 Patients

This retrospective observational study used prospectively collected data from a database of a single institution. Between January 2015 and May 2020, 534 consecutive patients with 803 lesions underwent ESD for early esophageal cancer under intravenous anesthesia without endotracheal intubation at our hospital. Patients who underwent ESD for multiple synchronous lesions on the same day, patients who underwent repeat ESD, patients after laryngectomies, and patients after esophagectomy were excluded. Propensity score analysis was performed to reduce the effects of selection bias for the use of an overtube and other possible confounding factors [11].

The ethics committee of Osaka City University Graduate School of Medicine approved the study protocol (number 2020‐025). Information about this study was available online, and the participants provided written informed consent.

### 2.2 Outcomes

To clarify the impact of overtube use on the incidence of aspiration pneumonia during esophageal ESD, the following outcomes were set: The primary outcome of this study was the risk factors for aspiration pneumonia during esophageal ESD. The secondary outcome was the incidence of aspiration pneumonia during esophageal ESD. Additionally, differences in the severity of aspiration pneumonia according to overtube use were evaluated.

### 2.3 ESD Procedure

**FIGURE 1:**
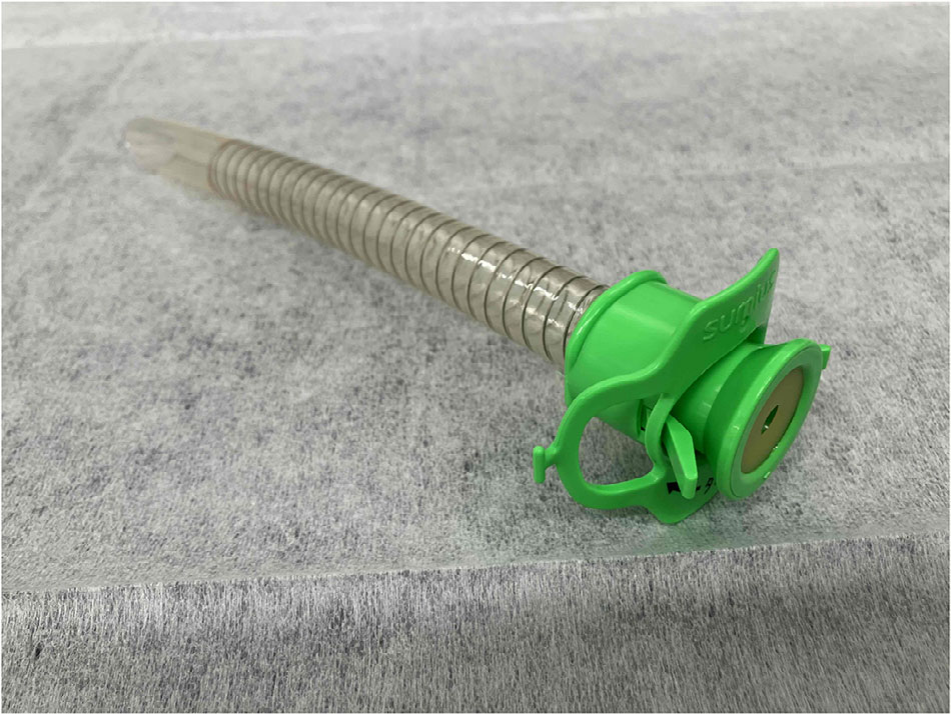
Flexible overtube (MD‐48518; Sumitomo Bakelite Co., Tokyo, Japan).

The patients were placed in the left lateral position. Sedation during ESD was performed using pethidine hydrochloride and midazolam or propofol, and the sedation level was controlled at moderate to deep sedation [10, 12]. A single‐channel endoscope with a water‐jet system (GIF‐Q260J or GIF‐290T; Olympus Medical Systems Co., Tokyo, Japan), transparent hood (D‐201‐11804; Olympus) attached to the tip of the endoscope, carbon dioxide insufflator (UCR; Olympus), and standard electrosurgical generator (VIO300D; Erbe Elektro Medizin GmbH, Tubingen, Germany) were used. A monopolar needle knife (Flush Knife; DK2618JN; Fujifilm Medical, Tokyo, Japan) was used. A Coagrasper (FD‐410LR; Olympus) or HemoStat‐Y (H‐S2518; Pentax Co, Tokyo, Japan) was used to stop bleeding or prevent hemorrhage before cutting the vessels. The use of a flexible overtube (MD‐48518: Sumitomo Bakelite Co, Tokyo, Japan) (Figure 1) during ESD was at the discretion of the endoscopists. An overtube was not used in patients with a history of chemoradiotherapy for laryngeal or cervical esophageal cancer or in those with severe stenosis in the overtube insertion route. In ESD for cervical or upper thoracic esophageal lesions, when insertion of the overtube to its full length resulted in the distal tip being positioned beyond the lesion on the distal side, the overtube was partially withdrawn and repositioned to an appropriate depth. However, an overtube was not used, particularly in cases of cervical lesions, where its use was technically difficult.

The ESD procedure has been previously described in detail as follows [13]: 1) marking around the lesion, 2) submucosal injection with a hyaluronic acid solution, 3) circumferential mucosal incision, and 4) submucosal dissection. None of the patients underwent endotracheal intubation for airway protection. The nurses positioned themselves behind the patients and periodically administered oral suction during the ESD procedure. Oxygen was administered when hypoxemia occurred at an oxygen saturation of <94%.

### 2.4 Definition

Aspiration pneumonia was defined as evidence of new pulmonary consolidation on chest radiographs or computed tomography (CT) with pulmonary symptoms, including cough or sputum [10, 14]. Chest radiographs and CT imaging were performed if there was obvious aspiration during ESD, if the SpO_2_ decreased, or if there was fever, cough, or sputum after the ESD. Endoscopists with ≤100 ESD cases were defined as non‐Expert, and those with >100 ESD cases were defined as Expert [15, 16].

### 2.5 Statistical Analyses

Data are presented as means (standard deviations) for continuous variables and as frequencies (percentages) for categorical variables. The variables were evaluated using unpaired t‐tests for continuous values and the chi‐squared test (or Fisher's exact test when necessary because of small sample sizes) for categorical values. A propensity score‐matched cohort was created by attempting to match each patient with a similar patient regarding whether or not an overtube was used during esophageal ESD (1:1 matching) using a matching technique to reduce the effects of selection bias and potential confounding factors. We used our clinical experience and knowledge to select possible confounders for their potential association with outcomes as follows: age, sex, American Society of Anesthesiologists Physical Status, lung disease, antithrombotic agents, smoking, history of radiation therapy for head and neck cancer, history of radiation therapy for esophageal cancer, location, endoscopic appearance, tumor diameter, circumference, histological type, invasion depth, previous ESD scarring, endoscopist experience, sedation, and traction method. After matching, crude comparisons of the matched cohorts were performed using the chi‐squared test and paired t‐tests. Covariate balance after propensity score matching was assessed using standardized mean differences (SMDs). The risk factors for aspiration pneumonia during esophageal ESD were estimated by calculating the odds ratio (OR) and 95% confidence interval (CI). In addition, inverse probability of treatment weighting (IPTW) analysis, which was used to adjust for confounders without reducing the sample size, was used to confirm that the results were qualitatively similar to those of the sensitivity analysis. Logistic regression analysis using IPTW was performed to analyze the risk factors of aspiration pneumonia during esophageal ESD. All statistical tests were two‐sided, and *p*‐values < 0.05 were considered statistically significant. Statistical analyses were performed using SPSS version 26.0 for Windows (SPSS, Tokyo, Japan).

## 3 Results

### 3.1 Patient Characteristics

**FIGURE 2:**
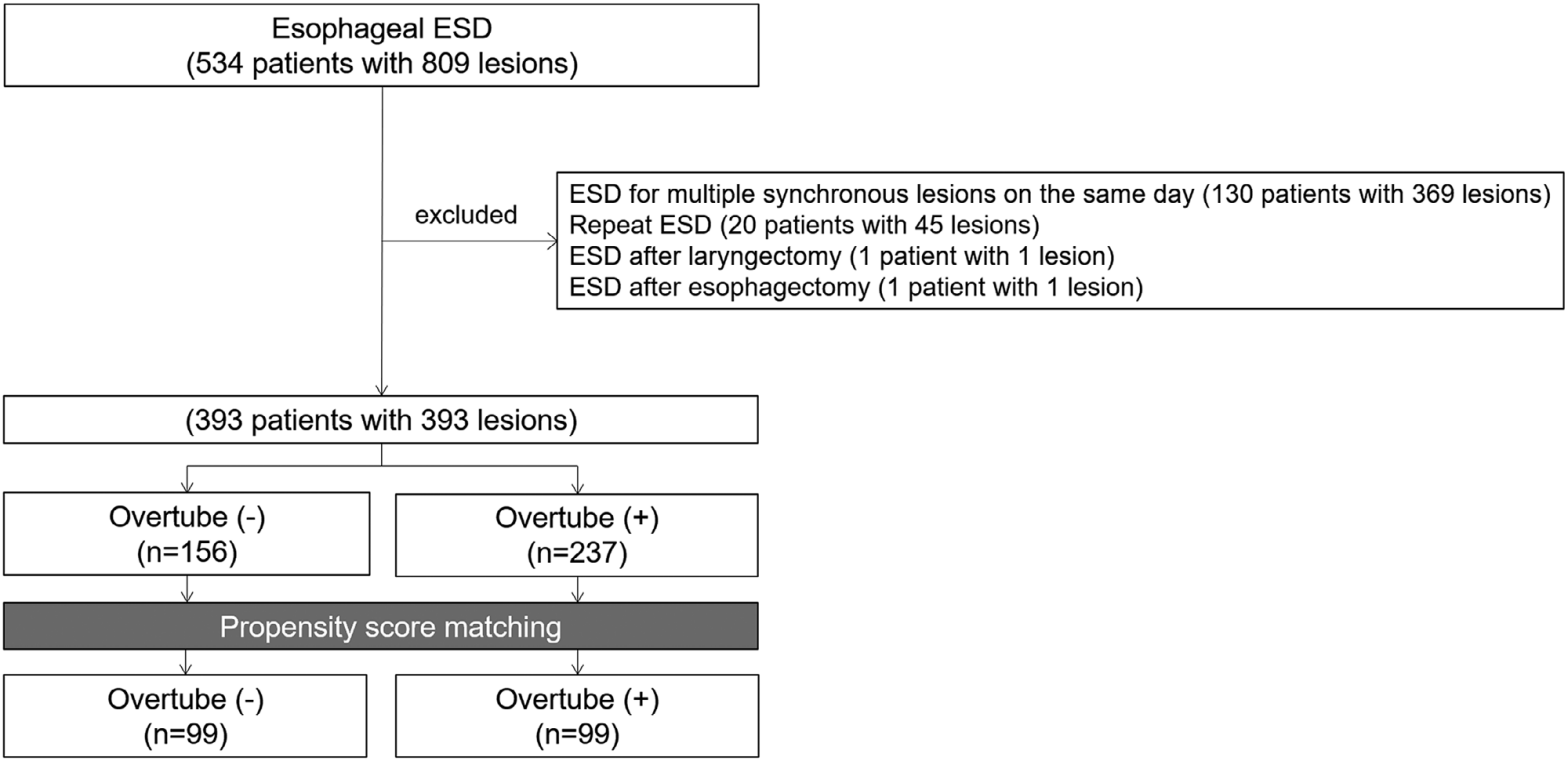
Diagram of the study design. ESD, endoscopic submucosal dissection.

Among the 534 consecutive patients with 803 lesions who underwent esophageal ESD, 131 patients with 369 lesions who underwent ESD for multiple synchronous lesions on the same day, 20 patients with 45 lesions who underwent repeat ESD, one patient with one lesion after a laryngectomy, and one patient with one lesion after an esophagectomy were excluded. Consequently, 393 patients with 393 lesions were included in the study (Figure 2). An overtube was used during esophageal ESD in 60.3% of the patients, with 237 and 156 patients in the overtube and non‐overtube groups, respectively. The characteristics of the study patients are shown in Table 1.

**TABLE 1:** Patient and lesion characteristics before and after propensity score matching.

		Before matching (*n* = 393)				After matching (*n* = 198)			
		Overtube (‐)	Overtube (+)	*p*‐Value	SMD	Overtube (‐)	Overtube (+)	*p*‐Value	SMD
		(*n* = 156)	(*n* = 237)			(*n* = 99)	(*n* = 99)		
Age	Years, mean ± SD	69.4 ± 8.5	70.9 ± 9.4	0.119	0.167	71.0 ± 8.3	69.8 ± 9.6	0.335	0.134
Sex	Female	34 (21.8%)	54 (22.8%)	0.818	0.024	27 (27.3%)	24 (24.2%)	0.626	0.069
	Male	122 (78.2%)	183 (77.2%)			72 (72.7%)	75 (75.8%)		
ASA‐PS classification	1,2	139 (89.1%)	213 (89.9%)	0.807	0.025	93 (93.9%)	90 (90.9%)	0.420	0.115
	3	17 (10.9%)	24 (10.1%)			6 (6.1%)	9 (9.1%)		
Respiratory diseases	No	146 (87.2%)	214 (90.3%)	0.333	0.121	90 (90.9%)	91 (91.9%)	0.800	0.036
	Yes	20 (12.8%)	23 (9.7%)			9 (9.1%)	8 (8.1%)		
Antithrombotic drug	No	126 (80.8%)	192 (81.0%)	0.952	0.006	80 (80.8%)	80 (80.8%)	1.000	0.000
	Yes	30 (19.2%)	45 (19.0%)			19 (19.2%)	19 (19.2%)		
Smoking	No	23 (14.7%)	50 (21.1%)	0.113	0.166	19 (19.2%)	19 (19.2%)	1.000	0.000
	Yes	133 (85.3%)	187 (78.9%)			80 (80.8%)	80 (80.8%)		
A history of radiotherapy for head and neck cancer	No	138 (88.5%)	230 (97.0%)	0.001	0.336	93 (93.9%)	94 (94.9%)	1.000	0.044
	Yes	18 (11.5%)	7 (3.0%)			6 (6.1%)	5 (5.1%)		
A history of radiotherapy for esophageal cancer	No	144 (92.3%)	231 (97.5%)	0.024	0.236	92 (92.9%)	95 (96.0%)	0.537	0.133
	Yes	12 (7.7%)	6 (2.5%)			7 (7.1%)	4 (4.0%)		
Location	Mt, Lt, Ae	105 (67.3%)	150 (65.8%)	0.757	0.084	86 (86.9%)	88 (88.9%)	0.663	0.062
	Ce, Ut	51 (32.7%)	78 (34.2%)			13 (13.1%)	11 (11.1%)		
Macroscopic type	Elevated, Flat	108 (75.5%)	150 (65.8%)	0.047	0.126	71 (71.7%)	70 (70.7%)	0.875	0.022
	Depressed	35 (24.5%)	78 (34.2%)			28 (28.3%)	29 (29.3%)		
Tumor size	mm, mean ± SD	19.7 ± 13.3	23.8 ± 16.7	0.007	0.272	19.4 ± 14.1	21.8 ± 14.5	0.247	0.168
Circumference	%, mean ± SD	25.3 ± 17.4	31.1 ± 22.7	0.005	0.287	25.1 ± 17.2	28.3 ± 20.5	0.236	0.169
Histological type	Adenocarcinoma	4 (2.6%)	25 (10.5%)	0.003	0.327	4 (4.0%)	8 (8.1%)	0.373	0.170
	SCC	152 (97.4%)	212 (89.51%)			95 (96.0%)	91 (91.9%)		
Invasion depth	Non‐SM	143 (91.7%)	210 (88.6%)	0.326	0.103	93 (93.9%)	92 (92.9%)	0.774	0.041
	SM	13 (8.3%)	27 (11.4%)			6 (6.1%)	7 (7.1%)		
Previous ESD scar	No	141 (90.4%)	222 (93.7%)	0.230	0.122	88 (88.9%)	92 (92.9%)	0.323	0.131
	Yes	15 (9.6%)	15 (6.3%)			11 (11.1%)	7 (7.1%)		
Endoscopist	Non‐expert	47 (30.1%)	90 (38.0%)	0.110	0.166	32 (32.3%)	35 (35.4%)	0.652	0.064
	Expert	109 (69.9%)	147 (62.0%)			67 (67.7%)	64 (64.6%)		
Sedation	Midazolam	24 (15.4%)	46 (19.4%)	0.308	0.106	18 (18.2%)	13 (13.1%)	0.328	0.139
	Propofol	132 (84.6%)	191 (80.6%)			81 (81.8%)	86 (86.9%)		
Traction method	No	57 (36.5%)	68 (32.5%)	0.407	0.168	38 (38.4%)	36 (36.4%)	0.769	0.042
	Yes	99 (63.5%)	169 (67.5%)			61 (61.6%)	63 (63.6%)		

Abbreviation: ASA‐PS, American Society of Anesthesiologists‐Physical Status; ESD, endoscopic submucosal dissection; SD, standard deviation; SMD, standardized mean difference.

Before propensity score matching, there was a significant difference between the two groups with respect to history of radiation therapy to the head and neck, a history of radiation therapy to the esophagus, location, endoscopic appearance, tumor diameter, circumference, and histological type. After propensity score matching, 99 matched pairs of patients did or did not undergo overtube placement during esophageal ESD. Several baseline characteristics showed imbalances before matching; however, the matching procedure substantially improved the covariate balance. After matching, all covariates had SMDs below 0.20, indicating acceptable balance between the two groups (Table 1).

### 3.2 Incidence of Aspiration Pneumonia

**FIGURE 3:**
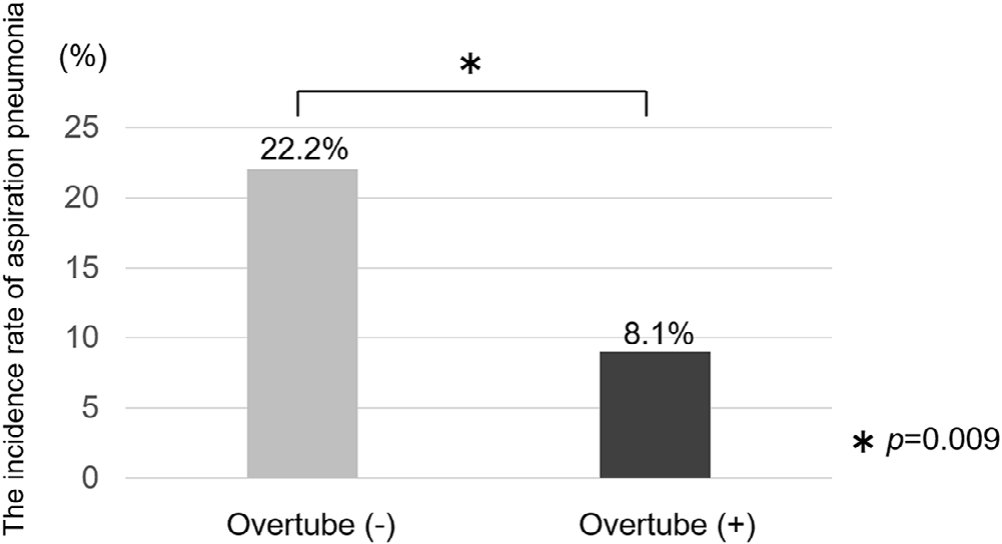
Incidence of aspiration pneumonia during esophageal endoscopic submucosal dissection.

After matching, the overall incidence of aspiration pneumonia was 15.2%. In 46.7% of these cases, aspiration pneumonia could be diagnosed based solely on chest radiography. The incidence of aspiration pneumonia was significantly higher in the non‐overtube group than in the overtube group (22.2% vs. 8.1%, *p* = 0.009) (Figure 3).

### 3.3 Risk Factors for Aspiration Pneumonia

Univariate logistic regression analysis revealed that the non‐use of an overtube (OR, 3.25; 95% CI, 1.37–7.71; *p* = 0.008), tumor diameter (OR, 1.04; 95% CI, 1.01–1.06; *p* = 0.002), circumference (OR, 12.20; 95% CI, 2.05–73.20; *p* = 0.006) and traction method (OR, 2.72; 95% CI, 1.06–7.01; *p* = 0.038) were risk factors for aspiration pneumonia (Table 2). Multivariate logistic regression analysis showed that non‐use of an overtube was an independent risk factor for aspiration pneumonia (OR, 4.21; 95% CI, 1.66–10.65; *p* = 0.002) (Table 3). After adjusting the model for differences in the baseline risk factors using the IPTW method, aspiration pneumonia was found to increase in association with non‐use of an overtube (OR, 3.76; 95% CI, 1.54–9.21; *p* = 0.004) (Table 4).

**TABLE 2:** The risk factors for aspiration pneumonia by univariate logistic regression analysis after propensity score matching.

		After matching (*n* = 198)			
		*n*	Case (%)	OR (95%CI)	*p‐*Value
Overtube	Yes	99	8 (8.1%)	1.00	
	No	99	22 (22.2%)	3.25 (1.37–7.71)	0.008
Age	Years, mean ± SD	198	30 (15.2%)	1.04 (0.99–1.09)	0.093
Sex	Female	51	12 (23.5%)	1.00	
	Male	147	18 (12.2%)	0.45 (0.20–1.02)	0.057
ASA‐PS classification	1, 2	183	26 (14.2%)	1.00	
	3	15	4 (26.7%)	2.20 (0.65–7.42)	0.205
Respiratory diseases	No	181	25 (13.8%)	1.00	
	Yes	17	5 (29.4%)	2.60 (0.84–8.01)	0.096
Antithrombotic drug	No	160	24 (15.0%)	1.00	
	Yes	38	6 (15.8%)	1.06 (0.40–2.81)	0.903
Smoking	No	38	7 (18.2%)	1.00	
	Yes	160	23 (14.4%)	0.74 (0.29–1.89)	0.533
A history of radiotherapy for head and neck cancer	No	187	29 (15.5%)	1.00	
	Yes	11	1 (9.1%)	0.55 (0.07–4.42)	0.570
A history of radiotherapy for esophageal cancer		187	28 (15.0%)	1.00	
		11	2 (18.2%)	1.26 (0.26–6.15)	0.773
Location	Mt, Lt, Ae	174	27 (15.5%)	1.00	
	Ce, Ut	24	3 (12.5%)	0.78 (0.28–2.79)	0.700
Macroscopic type	Elevated, Flat	141	22 (15.6%)	1.00	
	Depressed	57	8 (14.0%)	0.88 (0.37–2.12)	0.781
Tumor size	mm, mean ± SD	198	30 (15.2%)	1.04 (1.01–1.08)	0.002
Circumference	%, mean ± SD	198	30 (15.2%)	12.20 (2.05–73.20)	0.006
Histological type	Adenocarcinoma	12	2 (16.7%)	1.00	
	SCC	186	28 (15.1%)	0.89 (0.18–4.28)	0.880
Invasion depth	Non‐SM	185	27 (14.6%)	1.00	
	SM	13	3 (23.1%)	1.76 (0.46–6.78)	0.451
Previous ESD scarring	No	180	26 (14.4%)	1.00	
	Yes	18	4 (22.2%)	1.69 (0.52–5.54)	0.385
Endoscopist experience	Non‐expert	67	10 (14.9%)	1.00	
	Expert	131	20 (15.3%)	1.03 (0.45–2.34)	0.949
Sedation	Midazolam	31	3 (9.7%)	1.00	
	Propofol	167	27 (16.2%)	1.80 (0.51–6.35)	0.361
Traction method	No	74	6 (8.1%)	1.00	
	Yes	124	24 (19.4%)	2.72 (1.06–7.01)	0.038

Abbreviation: ASA‐PS, American Society of Anesthesiologists‐Physical Status; CI, confidence interval; ESD, endoscopic submucosal dissection; OR, odds ratio.

**TABLE 3:** Univariate and multivariate logistic odds ratios of aspiration pneumonia without overtube use after propensity score matching.

	OR (95%CI)	*p*‐Value
Unadjusted	3.25 (1.35–7.71)	0.008
Adjusted for tumor size	4.03 (1.61–10.09)	0.003
Adjusted for circumference	4.07 (1.61–10.12)	0.003
Adjusted for traction method	3.41 (1.42–8.18)	0.006
Adjusted for tumor size, circumference, and traction method	4.21 (1.66–10.65)	0.002

Abbreviation: CI, confidence interval; OR, odds ratio.

**TABLE 4:** Propensity score matched and weighted logistic odds ratio of aspiration pneumonia without overtube use.

	OR (95%CI)	*p*‐Value
After PS matching	3.25 (1.37–7.71)	0.008
IPTW	3.76 (1.54–9.21)	0.004

Abbreviation: CI, confidence interval; IPTW, Inverse probability of treatment weighting; OR, odds ratio; PS, propensity score.

### 3.4 Severity of Aspiration Pneumonia

The maximum white blood cell (WBC) count was significantly higher in the non‐overtube group than in the overtube group. There were no significant differences between the two groups regarding maximum body temperature, C‐reactive protein level, oxygen dosage, duration of antibiotic administration, or duration of hospitalization after ESD (Table S1).

## 4 Discussion

The incidence of aspiration pneumonia after esophageal ESD was 15.2%, and using propensity score analysis and the IPTW method, it was demonstrated that the non‐use of an overtube was a risk factor for aspiration pneumonia. This study indicated that the use of an overtube during esophageal ESD may prevent aspiration pneumonia.

The incidence of aspiration pneumonia during esophageal ESD has been reported to be 1.6%–32.0%, with a large variation [8, 9, 17]. This may be because of differences in the methods used to evaluate aspiration pneumonia among the studies. In particular, studies that evaluated aspiration pneumonia using CT showed a higher incidence of aspiration pneumonia; however, it is thought that they also detected minor pneumonia images that were not clinically problematic. In contrast, a large‐scale systematic review reported that the incidence of aspiration pneumonia after gastric ESD was 0.0%–14.4% [18]. The incidence of aspiration pneumonia is higher with esophageal ESD than with gastric ESD, likely because fluid is more likely to reflux into the oral cavity. To the best of our knowledge, no studies have investigated the risk factors for aspiration pneumonia after esophageal ESD. A large‐scale systematic review reported that risk factors for aspiration pneumonia during gastric ESD include older age, pulmonary disease, cerebrovascular disease, residual stomach, sedation with propofol, and long treatment times [18].

This study showed that not using an overtube increased the risk of aspiration pneumonia during esophageal ESD. Thus, using an overtube may prevent aspiration pneumonia during esophageal ESD. As the OR for aspiration pneumonia using the IPTW method was 3.71, the use of an overtube could prevent aspiration pneumonia by approximately one‐quarter. The use of an overtube during upper gastrointestinal ESD, involving, for example, the esophagus or stomach, has been reported to decrease the need for oropharyngeal suction [19]. This previous study involved a small number of cases, and no cases of aspiration pneumonia occurred; therefore, it was not possible to verify whether an overtube could prevent aspiration pneumonia. Another study has reported that lesions located in the esophagus and an amount of irrigation liquid of ≥1 L were independent risk factors for aspiration pneumonia during upper gastrointestinal ESD, involving, for example, the esophagus, stomach, or duodenum [20]. These reports suggest that because the esophagus is the closest to the larynx, which is the entrance to the trachea, the irrigation solution used during esophageal ESD may reflux into the oral cavity, making it more likely to cause aspiration pneumonia. The use of an overtube allows the irrigation fluid in the esophagus to be directly excreted from the body, reducing reflux into the oral cavity and leading to the prediction that aspiration pneumonia may be prevented. However, the preventive effects of the overtubes are not universal. Aspiration pneumonia may occur because of reflux into the mouth through the gap between the overtube and esophageal lumen, or owing to aspiration of oral secretions.

However, overtube‐related complications, such as transient vocal cord paralysis, cricopharyngeal perforation, proximal esophageal laceration, varix rupture, and free esophageal perforation, have also been reported [21]. Although no complications were associated with the use of an overtube in this study, caution is required while using an overtube. Additionally, for patients with a history of chemoradiotherapy for laryngeal or cervical esophageal cancer and for patients with severe stenosis in the insertion route, the use of an overtube may be restricted because of the risk of overtube‐related complications.

The severity of aspiration pneumonia was also compared according to overtube use. Only the maximum WBC count was significantly higher in the non‐overtube group. While this may indicate greater pneumonia severity without overtube use, WBC count measurement timing was not standardized, and the findings require confirmation. There are few reports on methods to prevent aspiration pneumonia during esophageal ESD. The use of continuous liquid‐suction catheter attachment in esophageal ESD reduced the volume of liquid reflux to the mouth and contributed to a decreased incidence of aspiration pneumonia on CT [9]. On the other hand, it has been reported that aspiration pneumonia did not occur during esophageal ESD under general anesthesia with tracheal intubation [17]. Some preventive measures are desirable, especially in cases with a high risk of aspiration pneumonia or where pneumonia is likely to become severe. The overtube is a simple and inexpensive device that can prevent aspiration pneumonia during esophageal ESD and is useful in clinical settings.

This study had several limitations. First, this was a small, single‐center, retrospective study; therefore, its statistical power may have been low. However, no previous studies have reported sample sizes similar to our study design. Propensity score analysis provides unbiased estimates, thereby reducing selection bias. Standard logistic regression analysis was used because the covariates were sufficiently balanced after matching. However, residual confounding and bias due to the matching design cannot be completely eliminated. Furthermore, because only 30 aspiration pneumonia events were observed, the possibility of model overfitting cannot be completely excluded despite the limited number of variables included in the multivariate model. Second, chest radiographs or CT were not performed immediately after ESD in all patients, and it is possible that some cases of aspiration pneumonia were caused by aspiration during recovery from sedation after ESD rather than by aspiration during ESD. Performing chest radiography or CT for all patients immediately after ESD is not appropriate, as it may result in the detection of pneumonia, which is not a clinical problem. Third, in the analysis of risk factors for aspiration pneumonia, we were limited to factors that could be assessed preoperatively; therefore, we were unable to evaluate the treatment period, which has been reported as a risk factor for aspiration pneumonia in gastric ESD [18]. Factors during ESD may have been significant risk factors and influenced the results of this study. Furthermore, because patients with multiple lesions treated on the same day were excluded from the analysis of the location of the lesions, it was not possible to evaluate whether multiple lesions were associated with aspiration pneumonia. Multiple lesions can also lead to an extended treatment duration, which may be a risk factor for aspiration pneumonia. Fourthly, an overtube was not used in some patients with histories of chemoradiotherapy for laryngeal or cervical esophageal cancer, or in patients with severe stenosis of the overtube insertion route, or in patients with cervical lesions. Therefore, there may have been a bias regarding the indications for using an overtube. Finally, the severity of aspiration pneumonia could not be adequately assessed because this study used retrospective data.

In conclusion, this study is the first to show that the non‐use of an overtube is a risk factor for aspiration pneumonia during esophageal ESD. Therefore, the use of an overtube may be considered to help prevent aspiration pneumonia. To validate our results and demonstrate the efficacy of the overtube in preventing aspiration pneumonia in esophageal cancer, a larger multicenter study is required.

## Author Contributions

**Study conception and design**: Masaki Ominami. **Data curation and acquisition**: Masaki Ominami, Shusei Fukunaga, Daiki Kitagawa, Mitsuhiro Kono, Shunsuke Takahashi, Tatsuya Kurokawa, Yoshinori Shimamoto, Yuki Hisaki, Natsumi Maeda, Yuki Ishikawa‐Kakiya, Kojiro Tanoue, and Akinari Sawada. **Manuscript preparation**: Masaki Ominami. **Writing – review and editing**: Shusei Fukunaga, Yu Nishida, Hirotsugu Maruyama, Yuji Nadatani, Koji Otani, and Shuhei Hosomi. **Final approval**: Yasuhiro Fujiwara. **Final approval of the article**: all authors

## Funding

The authors have nothing to report.

## Ethics Statement

The study protocol was approved by the ethics committee of Osaka City University Graduate School of Medicine (2020‐025).

## Consent

This study involved no invasive procedures or interventions, did not utilize any human‐derived biological samples, and was conducted solely using clinical information. In accordance with ethical guidelines, the study was implemented using an opt‐out approach.

## Conflicts of Interest

The authors declare no conflicts of interest.

## Supporting information




**Supporting File 1**: deo270400‐sup‐0001‐tableS1.docx
