# Supplementary material for: Efficacy of Overtube in Preventing Aspiration Pneumonia During Esophageal Endoscopic Submucosal Dissection
Source: DEN Open. 2026 Aug 11;7(1):e70400. doi: 10.1002/deo2.70400 (PMC13458985; doi:10.1002/deo2.70400)
Supplement: Supplementary file 1 [file DEO2-7-e70400-s001.docx]

**Supplementary Table 1. Severity of aspiration pneumonia**

|  |  | Overtube (–) | Overtube (+) | *p*-value |
| --- | --- | --- | --- | --- |
|  |  | (n=22) | (n=8) |  |
| Maximum body temperature | °C, mean ± SD | 37.7 ± 0.6 | 37.6 ± 0.81 | 0.782 |
| Maximum WBC count | /µL, mean ± SD | 14643.3 ± 4896.8 | 9926.7 ± 4473.5 | 0.003 |
| Maximum CRP level | mg/dL, mean ± SD | 5.03 ± 4.5 | 3.4 ± 3.5 | 0.184 |
| Maximum oxygen dosage | L/min, mean ± SD | 2.0 ± 1.5 | 1.7 ± 1.2 | 0.477 |
| Duration of antibiotic administration | days, mean ± SD | 5.4 ± 1.7 | 5.8 ± 1.7 | 0.437 |
| Duration of hospitalization after ESD | days, mean ± SD | 7.9 ± 4.8 | 8.0 ± 3.0 | 0.911 |
| WBC, white blood cell; CRP, C-reactive protein; ESD, endoscopic submucosal dissection; SD, standard deviation | | | | |
